# Sheehan Syndrome Unmasked by Adrenal Crisis Secondary to Severe Dengue Fever

**DOI:** 10.31486/toj.24.0019

**Published:** 2024

**Authors:** Ajay Kumar Mishra, Saboor Mateen, Firdaus Jabeen, Shivesh Singh, Pankaj Kumar Verma

**Affiliations:** Department of Medicine, Era's Lucknow Medical College and Hospital, Lucknow, Uttar Pradesh, India

**Keywords:** *Adrenal insufficiency*, *dengue*, *hypopituitarism*, *neuroendocrinology*, *postpartum hemorrhage*

## Abstract

**Background:** Sheehan syndrome is the infarction of a pituitary gland that has been physiologically enlarged as a result of postpartum bleeding. Agalactorrhea and amenorrhea are classic symptoms, but a constellation of manifestations occurs in both the acute and chronic forms. These manifestations can remain largely nonemergent unless Sheehan syndrome is complicated by severe adrenal dysfunction secondary to an inciting event such as dengue. We present a case of Sheehan syndrome that was uncovered in a patient with a dengue infection presenting as adrenal crisis.

**Case Report:** A 45-year-old female presented with symptoms of acute gastroenteritis and severe dehydration. Her medical history was significant for secondary amenorrhea for 14 years after her last delivery followed by symptoms of endocrine dysfunction. At presentation, the patient was in adrenal crisis with hypotension, hypoglycemia, and hyperthermia. Dengue nonstructural protein 1 antigen was positive, along with signs of plasma leakage. Bloodwork showed bicytopenia with abnormal liver enzymes. Ultrasonography and computed tomography of the abdomen were suggestive of serositis with acalculous cholecystitis. Magnetic resonance imaging of the brain revealed an empty sella. Anterior pituitary hormone levels were significantly decreased with low serum cortisol, and the patient's thyroid profile analysis suggested secondary hypothyroidism. The final diagnosis was Sheehan syndrome presenting as adrenal crisis precipitated by severe dengue fever. The patient was managed conservatively and discharged on hormone supplement therapy.

**Conclusion:** Sheehan syndrome is an important cause of panhypopituitarism in the developing world. Knowledge of Sheehan syndrome is important to help prevent its occurrence and reduce its resultant multifactorial effects.

## INTRODUCTION

Sheehan syndrome, also known as postpartum pituitary necrosis, is infarction of a physiologically enlarged pituitary gland (chiefly the anterior lobe) following significant postpartum bleeding; it manifests as either panhypopituitarism or selective hormonal insufficiency, with the former being more common.^1,2^ Sheehan syndrome occurs in up to 5 in 100,000 females,^3^ with a prevalence as high as 3.1% reported in India.^4^ Although advances in medical care have reduced the incidence of Sheehan syndrome, it continues to be a frequent cause of hypopituitarism in the developing world.^5^

Sheehan syndrome presents with a myriad of symptoms in both the acute and chronic forms, with varied latency and asymptomatic phases lasting from months to years. The most common acute presentation symptoms are agalactorrhea, irregular menses, amenorrhea, hypotension, tachycardia, hyponatremia, and hypoglycemia. Symptoms of chronic Sheehan syndrome include fatigue, weakness, hair loss, constipation, weight gain, disturbed attention span, cold intolerance, and bradycardia.^1^

Sheehan syndrome is an important cause of chronic secondary adrenal insufficiency, and the symptoms of secondary adrenal insufficiency can overlap with those of chronic Sheehan syndrome, including fatigue, weight loss, hypoglycemia, hyponatremia, and anemia. Patients with Sheehan syndrome may remain asymptomatic until the body is exposed to stresses such as surgery or infection, when the condition precipitates and presents as adrenal crisis. Symptoms of adrenal crisis are varied and include abdominal pain, nausea, vomiting, hyperthermia or hypothermia, hypotension, features of shock, hyponatremia, hyperkalemia, metabolic acidosis, low cortisol levels, loss of appetite, weight loss, diaphoresis, tachycardia, tachypnea, and hypoglycemia.^6,7^

Adrenal crisis may be the primary presentation in patients with undiagnosed adrenal insufficiency, as up to 50% of patients with adrenal crisis do not have a prior diagnosis of adrenal insufficiency.^8^

Dengue fever is an endemic viral disease in India that has varying signs and symptoms; one of the lesser-known manifestations is adrenal insufficiency.^9,10^

Sheehan syndrome unmasked by dengue fever is a rare presentation,^11-13^ and we report the case of a middle-aged female who presented with severe dengue fever and adrenal crisis that on further investigation was diagnosed as Sheehan syndrome.

## CASE REPORT

A 45-year-old female presented to the emergency department with complaints of high fever and chills for 4 days; abdominal pain, diarrhea, and vomiting for 2 days; and dizziness and extreme fatigue for 2 days. On initial presentation, the patient was febrile and in shock with a pulse rate of 112 beats per minute, blood pressure of 84/58 mm Hg, respiratory rate of 22 breaths per minute, temperature of 102 °F, and a blood oxygen saturation of 96% on room air. She was hypoglycemic, with a random plasma glucose level of 48 mg/dL (reference range, 70-200 mg/dL). General examination revealed pallor with sparse pubic and axillary hair. The remainder of the physical examination was unremarkable.

Further review of her history revealed major blood loss (approximately 2,000 mL) during her last delivery 14 years prior, followed by lactation failure and amenorrhea. In the years since her last pregnancy, the patient became anhedonic, with gradual weight loss, irritability, easy fatigability, and intermittent headaches. None of her symptoms was severe enough to require hospital admission.

Hematologic examination showed dimorphic anemia with thrombocytopenia. The remaining blood parameters were within normal limits, including leukocyte count and coagulation profile (Table). Biochemical assessment showed increased serum aspartate aminotransferase and alkaline phosphatase and abnormal thyroid function tests suggestive of secondary hypothyroidism. Kidney function tests were within reference ranges. Hormonal analysis indicated low levels of anterior pituitary hormones (growth hormone, adrenocorticotropic hormone, follicle-stimulating hormone, luteinizing hormone) and cortisol. The patient's urine and blood cultures were sterile. Dengue nonstructural protein 1 antigen test was positive.

**Table.** Laboratory Values on Admission

Laboratory Test	Value	Reference Range
**Complete blood count**		
Hemoglobin, g/dL	**8.6**	10-16.5
Total leukocyte count, cells/mm^3^	4,600	4,000-11,000
Platelet count, 10^9^/L	**90**	150-450
Reticulocyte count, %	0.8	0.2-2.0
Prothrombin time, seconds	12.6	10-13
International normalized ratio	1.11	0.6-1.5
Hematocrit, %	**25.1**	33-54
**Kidney function tests**		
Blood urea nitrogen, mg/dL	36	Females: 15.0-36.38
Creatinine, mg/dL	1.1	Females: 0.7-1.2
Serum sodium, mmol/L	140	135-145
Serum potassium, mmol/L	4.5	3.5-5.5
**Liver function tests**		
Total serum bilirubin, mg/dL	1.3	0.2-1.3
Direct bilirubin, mg/dL	0.1	0.0-0.3
Indirect bilirubin, mg/dL	1.2	0.2-0.8
Alanine aminotransferase, U/L	23	Females: ≤34
Aspartate aminotransferase, U/L	**60**	Females: Up to 31
Serum alkaline phosphatase, U/L	**252**	38-125
**Thyroid profile**		
Serum free T3, pmol/L	**<0.390**	2.0-4.4
Serum free T4, ng/dL	**0.224**	0.93-1.7
Serum thyroid stimulating hormone (ultrasensitive), mIU/L	3.11	0.54-5.3
Anti-thyroid peroxidase, IU/mL	0.80	≤5.61
**Pituitary/adrenal hormones**		
Growth hormone, ng/mL	**<0.05**	0-8
Adrenocorticotropic hormone (morning), pg/mL	**<5**	10-60
Prolactin (nonpregnant), ng/mL	3.9	Females: 3.0-18.6
Follicle-stimulating hormone (postmenopause), mIU/mL	**3.47**	23.0-116.3
Luteinizing hormone (postmenopause), IU/L	**1.24**	5.16-61.99
Cortisol (morning), μg/dL	**1.89**	6.24-18
Antidiuretic hormone, pg/mL	**23.6**	0-5.9
**Other tests**		
Plasma dopamine, pg/mL	**142.16**	0-30
Serum calcium, mg/dL	8.6	8.6-10.2
Total protein, g/dL	**6.3**	Adults: 6.5-8.3
Serum albumin, g/dL	**3.2**	Adults: 3.5-5.2
Serum lactate dehydrogenase, U/L	204	120-246
Serum vitamin B12, pg/mL	614	239-931
Serum ferritin, ng/mL	253	Females <50 years: 6.24-137
Total iron binding capacity, μg/dL	338	Females: 265-497
Iron, μg/dL	43	Females: 49-180
Transferrin saturation, %	**12.72**	15-50
Procalcitonin, ng/mL	0.047	<0.500
Hemoglobin A1c, %	5.6	Nondiabetic: <5.7

Note: Clinically significant values are in bold.

**Figure 1.**
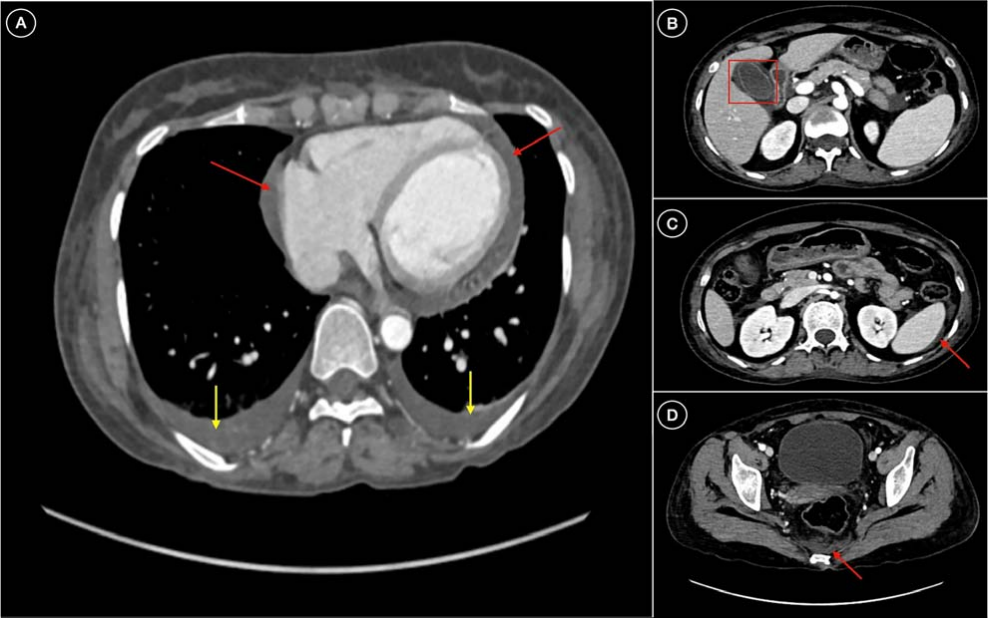
Axial contrast-enhanced computed tomography of the abdomen shows (A) pericardial effusion (red arrows) and bilateral pleural effusion (yellow arrows); (B) edematous gallbladder wall with surrounding pericholecystic fluid (acalculous cholecystitis) (red box); (C, D) minimal fluid in the pelvic region, perisplenic region, bilateral pararenal space, and adjacent bowel loops (red arrows).

**Figure 2.**
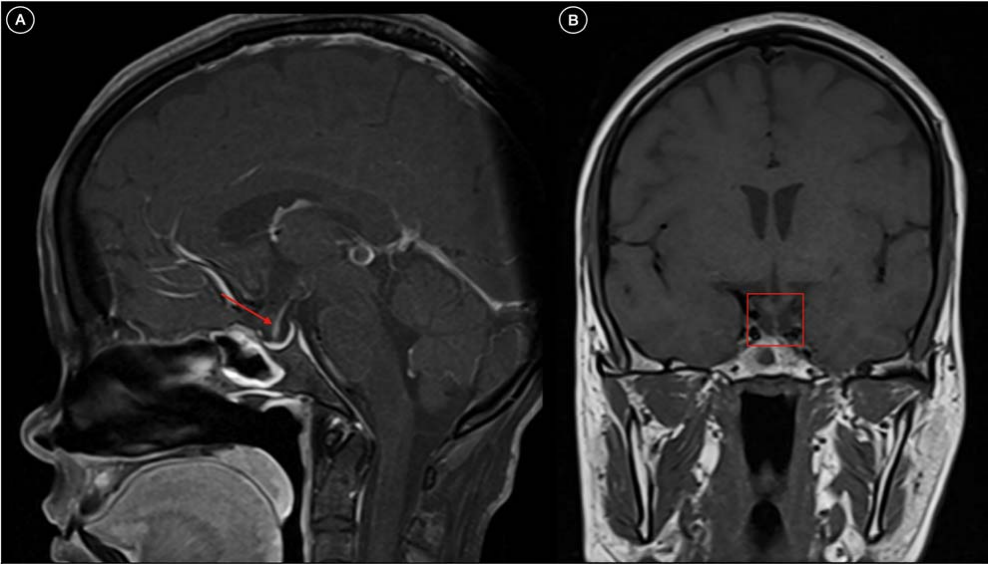
T1-weighted magnetic resonance imaging shows (A) a normal-appearing sella filled with cerebrospinal fluid with the infundibular stalk traversing to the floor of the sella (sagittal view; red arrow) and (B) an empty sella with the infundibular stalk extending to the base of the sella (coronal view; red box).

**Figure 3.**
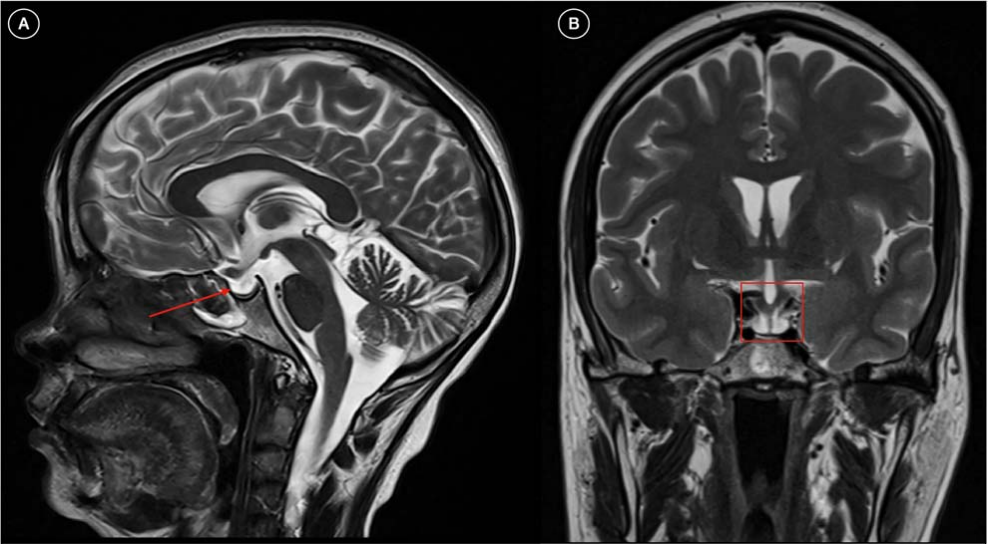
T2-weighted magnetic resonance imaging shows (A) a normal-appearing sella filled with cerebrospinal fluid (sagittal view; red arrow) and (B) the infundibular stalk traversing the empty cerebrospinal fluid–filled sella without displacement (coronal view; red box).

Ultrasonography of the abdomen showed ascites with acalculous cholecystitis. Contrast-enhanced computed tomography of the abdomen showed signs of plasma leakage with serositis (pleural effusion, pericardial effusion, and interbowel fluid collection) with acalculous cholecystitis (Figure 1) secondary to dengue infection. Magnetic resonance imaging of the brain with sella dynamic study revealed an empty sella filled with cerebrospinal fluid (Figures 2 and 3).

Based on the patient's medical history of severe postpartum uterine bleeding, more than 1 pituitary hormone deficiency, and an empty sella, the final diagnosis was Sheehan syndrome presenting as adrenal crisis precipitated by severe dengue fever.

Because of the adrenal crisis associated with severe dengue, fluid resuscitation was initiated, starting with an intravenous (IV) bolus dose of 1,000 mL of 0.9% of sodium chloride over 30 minutes, followed by further infusion at a rate of 20 mL/kg/h for 6 hours. The infusion rate was then reduced to 10 mL/kg/h, then to 5 mL/kg/h, and then to 3 mL/kg/h for the next 2 days. Afterward, fluid therapy was maintained at 2 mL/kg/h for the next 3 days. Pulse pressure was maintained at >20 mm Hg from day 3 onward. All forms of IV fluid maintenance were withdrawn on day 5 of admission.

Noradrenaline infusion of 12 μg/min was started on presentation, titrated to an average maintenance dose of 4 μg/min for the next 12 hours postadmission, and discontinued when the patient's blood pressure was maintained at >120/80 mm Hg continuously for >4 hours after 18 hours.

Because the patient's presenting plasma glucose was 48 mg/dL, she was also administered 200 mL of IV 10% glucose over 10 minutes on admission. Her glucose level improved to 100 mg/dL; however, she was maintained on a dextrose infusion of 100 mL/h for the next 24 hours. IV hydrocortisone was administered at a dose of 100 mg on admission, followed by 50 mg every 6 hours for 24 hours. Tapering doses of IV hydrocortisone were given at 6-hour intervals for the next 72 hours and overlapped with 20 mg/d of oral prednisolone.

The patient was discharged on day 10 of admission with resolution of abdominal pain, diarrhea, fever, and vomiting. Symptoms of fatigue and muscle weakness improved, and her blood pressure remained 120 to 129 mm Hg systolic and 80 to 84 mm Hg diastolic during the next 3 months of follow-up. Growth hormone replacement was started at 0.3 mg/d on discharge, and then titrated upward to 0.5 mg/d for the next 2 months and thereafter, resulting in maintained insulin-like growth factor 1 levels of 100 to 200 μg/L. Oral prednisolone was continued at a dose of 20 mg/d for 1 month. The patient's thyroid-stimulating hormone level at her 1-month follow-up continued to be within normal limits (0.54 to 5.3 mIU/L), and her serum free T3 and serum free T4 showed slight improvement (T3 of 0.41 pmol/L; T4 of 0.30 ng/dL). Prednisolone was continued at 20 mg/d for another 3 months (at the end of month 4, the patient's free T3 level had improved to 0.54 pmol/L, and her T4 level had also improved to 0.41 ng/dL) and then tapered to a 10 mg/d maintenance dose for 6 months with further endocrinology follow-up.

## DISCUSSION

Sheehan syndrome is an established cause of secondary adrenal insufficiency and is one of the most common precursors of panhypopituitarism occurring secondary to ischemic pituitary necrosis.^14^

The posterior pituitary receives blood directly from the inferior hypophyseal artery. The anterior pituitary is supplied indirectly from 2 sources: the long portal vessels that supply 70% of the blood to the gland and arise from the superior hypophyseal artery, and the short portal vessels that supply 30% of blood to the gland and arise from the posterior pituitary. These portal vessels are extremely sensitive to volume and pressure changes in the systemic circulation. The posterior pituitary is a higher pressure zone than the anterior pituitary because of the direct blood supply. Therefore, when pituitary apoplexy occurs as the result of low pressure or hypovolemia, the adenohypophysis (anterior pituitary) is affected as a whole, whereas the neurohypophysis (posterior pituitary) is generally preserved. During pregnancy, the anterior pituitary undergoes a 2- to 3-fold enlargement because of hyperplasia and hypertrophy of the lactotroph cells, increasing blood supply pressure. An associated postpartum hemorrhage as in Sheehan syndrome leads to an even more compromised state because of the infarction of an already physiologically enlarged pituitary.^1,15-18^

At least 75% of the pituitary must be infarcted before clinical manifestations become evident.^5^ The clinical manifestations of Sheehan syndrome involve some form of anterior lobe hormonal dysfunction.^19^ Growth hormone and prolactin level decreases are evident in 90% to 100% of cases, thyroid-stimulating hormone and adrenocorticotropic hormone decreases in 55% to 100% of cases, and follicle-stimulating hormone and luteinizing hormone deficiencies in 9% to 100% of cases.^5,16^

Our patient presented with a history of lactation failure following her last delivery that was associated with heavy postpartum bleeding because of uterine atony. Schrager and Sabo reported a similar case with agalactorrhea, one of the most common immediate indications of Sheehan syndrome.^20^ Following delivery, our patient's menses did not resume, and during the next 14 years, she developed the nonspecific symptoms of intermittent headache, easy fatigability, and loss of pubic and axillary hair. Her symptoms track with the Du et al study that reported amenorrhea (82.5%), agalactia (74.2%), and loss of pubic or axillary hair (85.6%) to be the most common clinical presentation in patients with Sheehan syndrome.^21^

The delayed diagnosis of Sheehan syndrome—14 years in our case—has also been reported by Gokalp et al.^22^ In their study of 124 patients, the mean interval between a complicated delivery and diagnosis of Sheehan syndrome was 20.37 ± 8.34 years.^22^

Studies suggest that hyponatremia is one of the most common laboratory manifestations of Sheehan syndrome, occurring in approximately 60% of acute cases and 33% to 69% of chronic cases.^23^ The mechanism is multifactorial: increased antidiuretic hormone release secondary to glucocorticoid deficit (subsequent loss of inhibition and increased hypothalamic secretion of corticotropin-releasing hormone), adrenal insufficiency, reduced cardiac output, and hypotension result in hyponatremia because of the reduced free water clearance.^23^ However, our patient's serum levels of sodium and potassium were within normal limits even though she presented in adrenal crisis (severe fatigue, weakness, abdominal pain, vomiting, dizziness, fever, hypotension, anemia, and hypoglycemia) with low cortisol and high antidiuretic hormone levels. Absence of hyponatremia despite adrenal crisis can be attributed to possible normal aldosterone levels (regulated primarily by the renin-angiotensin-aldosterone system independent of the hypothalamus and the pituitary^24^), as well as the relative preservation of the hypothalamus and some, if not all, of the posterior pituitary.

Recurrent symptomatic hypoglycemia is a reported manifestation of both acute and chronic Sheehan syndrome,^25,26^ and our nondiabetic patient presented with level 2 hypoglycemia (random plasma glucose level <54 mg/dL)^27^ but no severe autonomic symptoms, an unusual presentation that can be attributed to hypoglycemic unawareness resulting from chronic exposure to low blood glucose, recurrent severe hypoglycemic episodes, and the failure of counter-regulatory hormones.^28^

Lim et al suggested that dengue can cause panhypopituitarism, with pituitary infarction secondary to dengue shock syndrome–associated hypotension and blood pressure oscillations.^29^ Case reports show the existence of Sheehan syndrome in dengue patients,^11-13^ but few attribute dengue as a precursor of adrenal crisis in a patient with Sheehan syndrome as in our case. The mechanism by which severe dengue fever causes adrenal crisis and shock has been postulated to be the replication of the dengue virus within the adrenal gland.^30^

The cornerstone of treatment of Sheehan syndrome is the lifelong replacement of deficient hormones with a tailored approach such as our patient's discharge regimen and routine follow-up.

## CONCLUSION

The sequence of events—dengue as a possible precursor to adrenal crisis resulting in the revelation of Sheehan syndrome—makes our case unique. Sheehan syndrome can be a complication of postpartum hemorrhage and is most common in the developing world. The chronic form of Sheehan syndrome remains largely undiagnosed and manifests during acute insults when the production of counter-regulatory hormones is insufficient. Awareness of Sheehan syndrome and familiarity with its presenting symptoms can help ensure adequate treatment before adrenal crisis ensues.

## Notes

*This article meets the Accreditation Council for Graduate Medical Education and the American Board of Medical Specialties Maintenance of Certification competencies for Patient Care and Medical Knowledge.*
